# Determination of 37 fentanyl analogues and novel synthetic opioids in hair by UHPLC-MS/MS and its application to authentic cases

**DOI:** 10.1038/s41598-020-68348-w

**Published:** 2020-07-14

**Authors:** Nan Qin, Min Shen, Ping Xiang, Di Wen, Baohua Shen, Hongxiao Deng, Huosheng Qiang, Fenyun Song, Yan Shi

**Affiliations:** 1 Department of Forensic Toxicology, Shanghai Key Laboratory of Forensic Medicine, Shanghai Forensic Science Platform, Academy of Forensic Science, Shanghai, 200063 China; 2 School of Pharmacy, Guangdong Pharmaceutical University, Guangzhou, 510006 China; 3 College of Forensic Medicine, Hebei Medical University, Hebei Key Laboratory of Forensic Medicine, Collaborative Innovation Center of Forensic Medical Molecular Identification, Shijiazhuang, 050000 China

**Keywords:** Bioanalytical chemistry, Mass spectrometry

## Abstract

The recent emergence of new fentanyl analogues and synthetic opioids on the drug market poses a global public health threat. However, these compounds cannot typically be identified using existing analytical methods. In this study, we aimed to develop and validate a rapid and sensitive method based on ultra-high-performance liquid chromatography–tandem mass spectrometry (UHPLC-MS/MS) for the simultaneous determination of 37 fentanyl analogues and novel synthetic opioids in hair samples. Hair samples (20 mg) were extracted by cryogenic grinding in an extraction medium of methanol, acetonitrile, and 2 mmol/L ammonium acetate (pH 5.3). Following centrifugation of the samples, the analytes were separated using a WATERS Acquity UPLC HSS T_3_ column. The limits of detection (LODs) and limits of quantification (LOQs) ranged from 0.5 to 2.5 pg/mg and from 2 to 5 pg/mg, respectively. The intraday and interday precisions were within 13.32% at LOQ, low, medium, and high levels. The accuracies were within the range of 85.63–116.1%. The extraction recoveries were in the range of 89.42–119.68%, and the matrix effects were within the range of 44.81–119.77%. Furthermore, the method was successfully applied to the detection and quantification of fentanyl and sufentanil in hair samples from two authentic cases. Thus, this method has great potential for detecting fentanyl analogues and novel synthetic opioids in forensic work.

## Introduction

Recently, new psychoactive substances (NPSs) have emerged in illicit markets^1,2^. Although many countries are trying to curb this trend, e.g., more than 170 NPSs are currently controlled in China, NPSs still pose a threat to public health. Most NPSs are synthetic cannabinoids and designer cathinones, but there has been a sharp increase in the consumption of novel synthetic opioids, particularly fentanyl and its analogues^3^. Fentanyl, a μ-opioid receptor agonist, is used as an analgesic and anesthesia adjuvant with a 50–100 times higher potency than morphine^4–6^. However, numerous illicit fentanyl substances have been used to adulterate other abuse drugs such as heroin, cocaine, and methamphetamine. Fentanyl and its analogues, which have high potency and numerous side effects, e.g., respiratory depression, have caused many fatalities^7^. This problem is further aggravated by the easy availability of these substances via complex international networks and their use with other drugs^6,8,9^. Daniulaityte et al.^10^ reported that in Montgomery County (located in southeast Ohio), the number of unintentional overdose death cases in which positive tests for fentanyl analogues were obtained increased by 337% between the second half of 2015 and the first half of 2017.

As a consequence of increasing fatalities and the emergence of new fentanyl analogues and novel synthetic opioids, forensic laboratories must update their analytical methods for the identification and quantification of these drugs in various biological matrices. Some methods have been developed for the detection of these substances in whole blood^11–13^, urine^14^, saliva^15–17^, and dried blood spots^18,19^. However, it has been reported that the stability of some fentanyl analogues in whole blood is one month or less^20,21^. In other words, fentanyl analogues have a short period of storage time in such samples. Moreover, fentanyl is metabolized quickly in vivo, as the half-life of fentanyl in adults is 3.7 h and in infants is 5.3–21.1 h^22^. Therefore, to supplement these methods, it is necessary to develop methods for analyzing hair samples.

Hair, as an alternative or complementary matrix to conventional matrices, is a research hotspot in forensic science. Hair analysis can provide a longer detection window because substances may remain in hair for a long time without significant degradation. Furthermore, hair is easy to access, transport, and store^23,24^. To the best of our knowledge, only a few comprehensive methods using LC–MS/MS have been reported for analyzing fentanyl analogues and novel synthetic opioids in hair^3,25^. However, only a limited number of compounds were included in these methods.

Despite the advantages of hair analysis, the drug concentration in hair after a single use is usually on the pg/mg level^26^. Moreover, as fentanyl analogues typically have low active concentration^27^, higher sensitivity is required for the analysis of fentanyl analogues in hair. Compared with other methods, such as gas chromatography with nitrogen–phosphorous detection^28^ and gas chromatography–mass spectrometry^29^, liquid chromatography–tandem mass spectrometry^30,31^ is promising for analyzing fentanyl and its analogues in hair owing to its short analysis time, selectivity, and sensitivity.

In this study, an ultra-high-performance liquid chromatography–tandem mass spectrometry (UHPLC-MS/MS) method was developed and validated for the identification and quantification of 31 fentanyl analogues and 6 novel synthetic opioids in hair. Furthermore, the developed method was successfully applied to authentic cases.

## Materials and methods

### Chemicals and regents

Standards of fentanyl, norfentanyl, alfentanil, acetyl norfentanyl, U-47700, *N*-desmethyl U-47700, U-48800, U-50488, and W-18 (containing 1 mg/mL free base); 4-anilino-*N*-phenethylpiperidine (4-ANPP), norcarfentanil, acryl fentanyl, butyryl fentanyl, isobutyryl fentanyl, *para/ortho*-fluorofentanyl, *para*-fluorobutyryl fentanyl (PFBF), 4-fluoroisobutyryl fentanyl, *cis*-3-methylfentanyl, *β*-hydroxythiofentanyl, valeryl fentanyl, ocfentanil, furanyl fentanyl, sufentanil, remifentanil, carfentanil, cyclopropylfentanyl, and methoxyacetylfentanyl (containing 100 μg/mL free base); and acetyl fentanyl (containing 50 μg/mL free base) were obtained as methanol or acetonitrile solutions from CERILLIANT (Round Rock, TX, USA). 3-Methylthiofentanyl, *trans*-3-methylfentanyl, *α*-methylfentanyl, *β*-hydroxyfentanyl, *β*-hydroxy-3-methylfentanyl, thiofentanyl, and tetrahydrofuranyl fentanyl (THF-F) (1 mg/mL) were synthesized by the Criminal Investigation Department of the Shanghai Public Security Bureau (Shanghai, China). The deuterated internal standards (ISs) of fentanyl-*d*_5_ (used for most fentanyl analogues), norfentanyl-*d*_5_ (used for norfentanyl, acetyl norfentanyl, and norcarfentanil), and U-47700-*d*_3_ (used for the novel synthetic opioids) were purchased from Cerilliant.

High-performance liquid chromatography (HPLC)-grade methanol and acetonitrile were obtained from SIGMA-SLDRICH (St. Louis, MO, USA). Formic acid (98%), ammonium acetate (98%), and ammonium formate (98%) were obtained from FLUKA (Seelze, Germany). Ultrapure water was produced using a Milli-Q system (MERCK, Darmstadt, Germany).

### Solution preparation

Working solutions were obtained by diluting their single solutions in one methanol mixture, and further dilutions of this mixture in methanol. Working solutions were prepared at 10 different concentration levels (4, 10, 20, 100, 200, 400, 1,000, 2,000, 2,500, and 4,000 ng/mL).

An IS mixture (10 ng/mL) was obtained by spiking the extraction medium (EM), which consisted of methanol, acetonitrile, and 2 mmol/L ammonium formate (25:25:25, v/v/v, pH 5.3) with 250 μL of a mixture (100 ng/mL) of fentanyl-*d*_5_, norfentanyl-*d*_5_, and U-47700-*d*_3_^32^.

### Hair specimens

Blank hair samples, provided voluntarily by the laboratory staff, were used for spiked calibration standards and quality control (QC) samples. The real hair samples used were from suspected users who were arrested and investigated by police. All the samples were stored at room temperature until analysis. All participants provided written informed consent and all study protocols were approved by the Ethics Committee of Academy of Forensic Science, Shanghai, China.

### Sample extraction

To remove contaminants, the samples were washed with water two times and acetone three times, and then air-dried at room temperature. The washed samples were cut into 2–3 mm pieces and weighed (20 mg) in 2 mL tubes. Then, ceramic beads and 1 mL of the EM (containing 10 ng/mL IS) were added to tube. Subsequently, the hair samples were extracted by cryogenic grinding using a Bead Ruptor system (OMNI, Kennesaw, GA, USA) at a speed of 6 m/s for 20 s and then allowed to cool for 40 s. This process was repeated 10 times. The pulverized samples were centrifuged for 3 min at 14,000 × *g* and filtered (pore size 0.22 μm). Finally, 200 μL of filtrate was transferred into an autosampler vial.

### UHPLC-MS/MS conditions

The UHPLC-MS/MS analysis was performed on an Acquity UPLC system (Milford, MA, WATERS, USA) coupled to a QTRAP 6500 PLUS triple quadruple linear ion trap mass spectrometer (AB SCIEX, Framingham, MA, USA). Sample separation was performed using on a WATERS Acquity UPLC HSS T_3_ column (100 mm × 2.1 mm, 1.8 μm) fitted with a 1.8 μm HSS T_3_ guard column. The mobile phase was composed of 20 mmol/L ammonium acetate solution containing 0.1% formic acid (mobile phase A) and acetonitrile (mobile phase B). The temperature of the autosampler was set at 4 °C and the injected volume was 5 μL. The gradient elution procedure is shown in Table 1.

**Table 1:** Steps of gradient elution.

Step	Time (min)	Flow rate (mL/min)	Mobile phase A (%)	Mobile phase B (%)
1	0	0.2	85.0	15.0
2	1.0	0.2	85.0	15.0
3	4.0	0.2	72.0	28.0
4	5.0	0.2	72.0	28.0
5	10.0	0.2	70.0	30.0
6	13.0	0.2	55.0	45.0
7	13.5	0.2	5.0	95.0
8	14.5	0.2	5.0	95.0
9	15.0	0.2	85.0	15.0
10	16.0	0.2	85.0	15.0

The mass spectrometer was equipped with an electrospray interface operating in positive ionization mode. The source temperature and ion spray voltage were set to 500 °C and 5,500 V, respectively. The gas parameters were set as follows: collision-activated dissociation (CAD) gas, medium; curtain gas (CUR), 30 psi; nebulizing gas, 40 psi; and heater gas, 40 psi. Detection was performed using multiple reaction monitoring (MRM) with two transitions for each analyte and IS. The first transition was used for quantification and the second for qualification. The MRM transitions and optimized mass spectrometric parameters for each compound are listed in Table 2.

**Table 2:** MRM transitions and mass spectrometric parameters for analytes and internal standards.

Analyte	Precursor ion (*m/z*)	Product ion (*m/z*)	Collision energy (eV)	Retention time (min)	Ion ratio	Internal standard
Fentanyl	337.2	188.3	35	8.48	0.38	Fentanyl-*d*_5_
	337.2	104.9	51			
Norfentanyl	233.1	84.0	29	3.93	0.27	Norfentanyl-*d*_5_
	233.1	55.2	44			
Alfentanil	417.3	268.3	25	7.87	0.69	Fentanyl-*d*_5_
	417.3	197.1	35			
Acetyl fentanyl	323.2	188.1	35	6.41	0.43	Fentanyl-*d*_5_
	323.2	105.0	50			
Acetyl norfentanyl	219.3	84.0	24	2.40	0.13	Norfentanyl-*d*_5_
	219.3	56.0	40			
4-ANPP	281.1	188.1	23	9.72	0.54	Fentanyl-*d*_5_
	281.1	105.1	40			
Acryl fentanyl	335.5	188.2	29	8.16	0.43	Fentanyl-*d*_5_
	335.5	105.0	41			
Butyryl fentanyl	351.3	188.1	29	10.84	0.37	Fentanyl-*d*_5_
	351.3	105.2	45			
Isobutyryl fentanyl	351.1	188.0	35	10.45	0.43	Fentanyl-*d*_5_
	351.1	105.0	60			
PFBF	369.2	188.2	30	12.20	0.27	Fentanyl-*d*_5_
	369.2	104.9	50			
4-Fluoroisobutyryl fentanyl	369.3	188.0	35	11.84	0.29	Fentanyl-*d*_5_
	369.3	105.0	60			
*para/ortho*-Fluorofentanyl	355.2	188.2	35	9.65	0.28	Fentanyl-*d*_5_
	355.2	104.9	50			
*β*-Hydroxythiofentanyl	359.3	191.9	34	6.05	0.51	Fentanyl-*d*_5_
	359.3	146.1	32			
*β*-Hydroxy-3-methylfentanyl	367.2	200.1	34	8.75	0.92	Fentanyl-*d*_5_
	367.2	218.1	31			
*β*-Hydroxyfentanyl	353.4	204.3	30	6.37	0.70	Fentanyl-*d*_5_
	353.4	186.0	33			
*cis*-3-Methylfentanyl	351.2	202.2	32	10.43	0.54	Fentanyl-*d*_5_
	351.2	105.2	52			
*trans*-3-Methylfentanyl	351.3	202.2	31	10.00	0.60	Fentanyl-*d*_5_
	351.3	105.1	55			
*α*-Methylfentanyl	351.3	202.0	30	9.41	0.26	Fentanyl-*d*_5_
	351.3	119.2	35			
3-Methylthiofentanyl	357.2	208.0	30	9.58	0.54	Fentanyl-*d*_5_
	357.2	111.0	50			
Thiofentanyl	343.0	194.0	30	7.52	0.24	Fentanyl-*d*_5_
	343.0	111.0	50			
Furanyl fentanyl	375.3	188.1	27	9.44	0.32	Fentanyl-*d*_5_
	375.3	105.2	50			
THF-F	379.3	188.2	32	6.62	0.29	Fentanyl-*d*_5_
	379.3	105.1	60			
Ocfentanil	371.2	188.2	32	6.39	0.30	Fentanyl-*d*_5_
	371.2	105.1	56			
Sufentanil	387.3	238.2	26	13.02	0.30	Fentanyl-*d*_5_
	387.3	355.3	25			
Remifentanil	377.3	228.3	26	5.52	0.79	Fentanyl-*d*_5_
	377.3	112.9	40			
Remifentanil acid	363.3	247.3	30	4.40	0.92	Fentanyl-*d*_5_
	363.3	112.9	41			
Carfentanil	395.1	335.1	26	11.46	0.38	Fentanyl-D_5_
	395.1	246.1	30			
Norcarfentanil	291.0	142.2	23	4.53	0.59	Norfentanyl-*d*_5_
	291.0	113.3	40			
Valeryl fentanyl	365.4	188.3	34	13.88	0.29	Fentanyl-*d*_5_
	365.4	105.2	60			
Methoxyacetylfentanyl	353.2	188.2	30	5.95	0.31	Fentanyl-*d*_5_
	353.2	105.2	55			
Cyclopropylfentanyl	349.1	188.2	32	9.87	0.29	Fentanyl-*d*_5_
	349.1	105.1	55			
U-47700	328.9	173.0	48	8.06	0.71	U-47700-*d*_3_
	328.9	203.9	37			
*N*-Desmethyl U-47700	315.0	204.0	35	7.47	0.65	U-47700-D_3_
	315.0	172.8	45			
U-50488	369.1	298.2	27	12.64	0.23	U-47700-*d*_3_
	369.1	218.0	40			
U-51754	343.0	218.1	36	9.68	0.76	U-47700-*d*_3_
	343.0	112.0	40			
U-48800	343.0	218.0	37	8.83	0.56	U-47700-*d*_3_
	343.0	112.1	42			
W-18	422.0	273.2	32	15.23	0.65	U-47700-*d*_3_
	422.0	111.0	70			
Fentanyl-*d*_5_	342.1	188.1	53	8.20		
	342.1	105.2	35			
Norfentanyl-*d*_5_	238.1	84.0	20.0	3.87		
	238.1	55.9	45			
U-47700-*d*_3_	331.9	287.0	25	7.90		
	331.9	207.1	37			

### Method validation

The method was developed according to the Society of Hair Testing (SoHT)^33^ guidelines and several recent criteria^34–36^ for method validation. Furthermore, the recovery and matrix effect (ME) were evaluated as described by Matuszewski et al*.*^37^.

#### Selectivity

The method selectivity was assessed using eight different sources of blank hair and spiking with the IS (10 ng/mL) to evaluate potential interference. Moreover, interference from possible coadministered medications was investigated according to our previous procedure^38^.

#### Limits of detections (LODs) and limits of quantification (LOQs)

To determine the LODs and LOQs, blank hair samples were spiked with analyte concentrations of 5.0, 2.5, 1.0, and 0.5 pg/mg, and three replicates of each concentration were analyzed. The concentration that gave a signal-to-noise (S/N) ratio greater than 3 for both the MRM transitions was chosen as the LOD. The LOQ was defined as the lowest calibration point with a coefficient of variation (CV) of less than 20% for the precision and accuracy in the range of 80–120%.

Calibration standards (2.0–2,500 pg/mg) were prepared by adding the working solutions to 20 mg of blank hair. In addition, QC samples were prepared at four concentration levels: LOQ (2 and 5 pg/mg), low (10 pg/mg), medium (500 pg/mg), and high (2,000 pg/mg). To determine linearity, seven sets of calibrators (two replicates for each set) were analyzed. The calibration curves were constructed by plotting the peak area ratios between each analyte and IS versus the concentration using 1/x weighting.

#### Accuracy and precision

The method precision and accuracy were assessed by analyzing spiked blank hair samples at four QC levels (LOQ, low, medium, and high). The precision was expressed as the CV. The intraday and interday precision were determined by analyzing six replicates on one day (*n* = 6) and over four days (*n* = 24), respectively. The CV for the precision should not exceed 15% for the low, medium, and high samples, whereas that for the LOQ sample should not exceed 20%. The accuracy was determined as the percentage ratio of the measured and theoretical values.

#### Recovery and ME

According to the method recommended by Matuszewski et al.^37^, the extraction recovery and ME were assessed at low (10 pg/mg), medium (500 pg/mg), and high (2000 pg/mg) levels. Hair samples from six drug-free individuals were used. For each level, the samples were divided into three groups (sets 1, 2, and 3). Set 1 consisted of neat standard solutions containing all the analytes in the EM. Set 2 was obtained by extracting the blank hair samples of six individuals and then spiking with the analytes. Set 3 was obtained by extracting the spiked hair samples using the method described in Sect. Sample extraction. The extraction recovery was calculated as the percentage ratio of the peak area of set 3 to the peak area of the set 2. The ME was defined as the percentage ratio of the peak area of set 2 to the peak area of set 1.

#### Stability

The stability of each analyte in hair was determined by injecting the extracted samples at three levels (*n* = 6) after storage in the autosampler at 4 °C for 24 h.

### Ethics approval and consent to participate

The hair collection was carried out in accordance with SoHT guidelines. All participants provided written informed consent and all study protocols were approved by the Ethics Committee of Academy of Forensic Science, Shanghai, China.

## Results and discussion

### Method development

#### Chromatographic conditions

**Figure 1:**
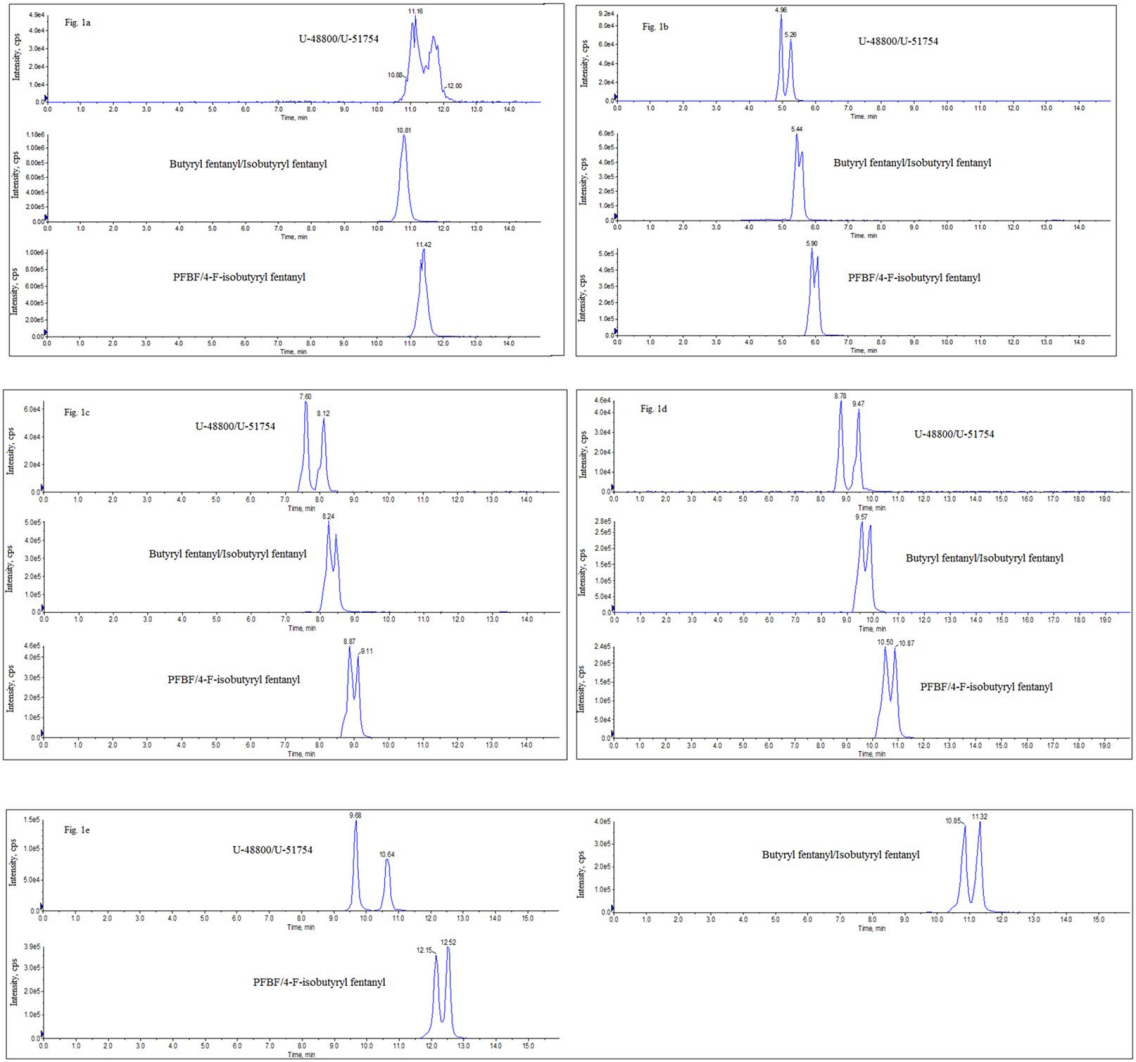
Chromatograms under different liquid conditions. (**a**) Different columns-WATERS T_3_ column; (**b**) different organic mobile phase-acetonitrile; (**c**) extending analysis time; (**d**) modifying the gradient elution; (**e**) reducing the flow rate.

In general, screening methods for fentanyl analogues requires must address the separation of isomers, e.g., PFBF and 4-fluoroisobutyryl fentanyl. Fogarty et al*.*^39^ has reported a method for detecting 18 fentanyl analogues in whole blood and separating three pair of isomers (butyryl fentanyl and isobutyryl fentanyl, *para*-fluorofentanyl and *ortho*-fluorofentanyl, and *β*-methylfentanyl and *α*-methylfentanyl). In our study, 31 fentanyl analogues including 5 pairs of isomers were analyzed. To separate the isomers, we optimized the gradient elution based on previous studies^13^. First, we compared analyte separation using a WATERS T_3_ column and a RESTEK PPFP column (100 × 2.1 mm, 5 μm) and found that better separation was achieved using the former column (Fig. 1a).Furthermore, as the method for isomer separation in the previous study took a long time (30 min), the T_3_ column was still used^13^. Based on other previous reports^39,40^, we used methanol instead of acetonitrile and found that the solvent did not influence the separation of the chromatographic peaks significantly (Fig. 1b). With the previous method^13^, the gradient elution time was extended to achieve isomer separation. As shown in Fig. 1c, this method cannot separate the isomers. Following refinement of the gradient, the separation was still not ideal as shown in Fig. 1d. Therefore, we reduced the flow rate from 0.3 to 0.2 mL/min, which allowed separation of four pairs of isomers, but not *para*-fluorofentanyl and *ortho*-fluorofentanyl (Fig. 1e).

#### Sample extraction conditions

The sample preparation conditions were also optimized. Methanol is typically selected as the extraction solvent for hair samples in previously methods for quantifying fentanyl analogues^3,41,42^. However, the EM has also been used to extract analytes from hair samples^32^. Hence, methanol and the EM were compared as extraction solvents in our study. The chromatographic behavior of the compounds was better when EM was used as the extraction solvent, especially for the isomers.

Subsequently, extraction using different volumes (500, 800, and 1,000 μL) of the EM was investigated. The recoveries of all compounds were in the range of 84.34–96.08%, 89.10–108.63%, and 83.27–104.15% with volumes of 500, 800, and 1,000 µL, respectively, with ME values in the range of 358.40–504.59%, 79.49–115.06%, and 77.92–104.30%, respectively. The EM volume had a great impact on the ME. In particular, when the analytes were extracted with 500 μL of the EM, the ME value increased significantly. However, for the recovery, the effect of the EM volume was not significant. Finally, the extraction solution was EM and the volume was 1,000 μL.

### Method validation

#### Selectivity

**Figure 2:**
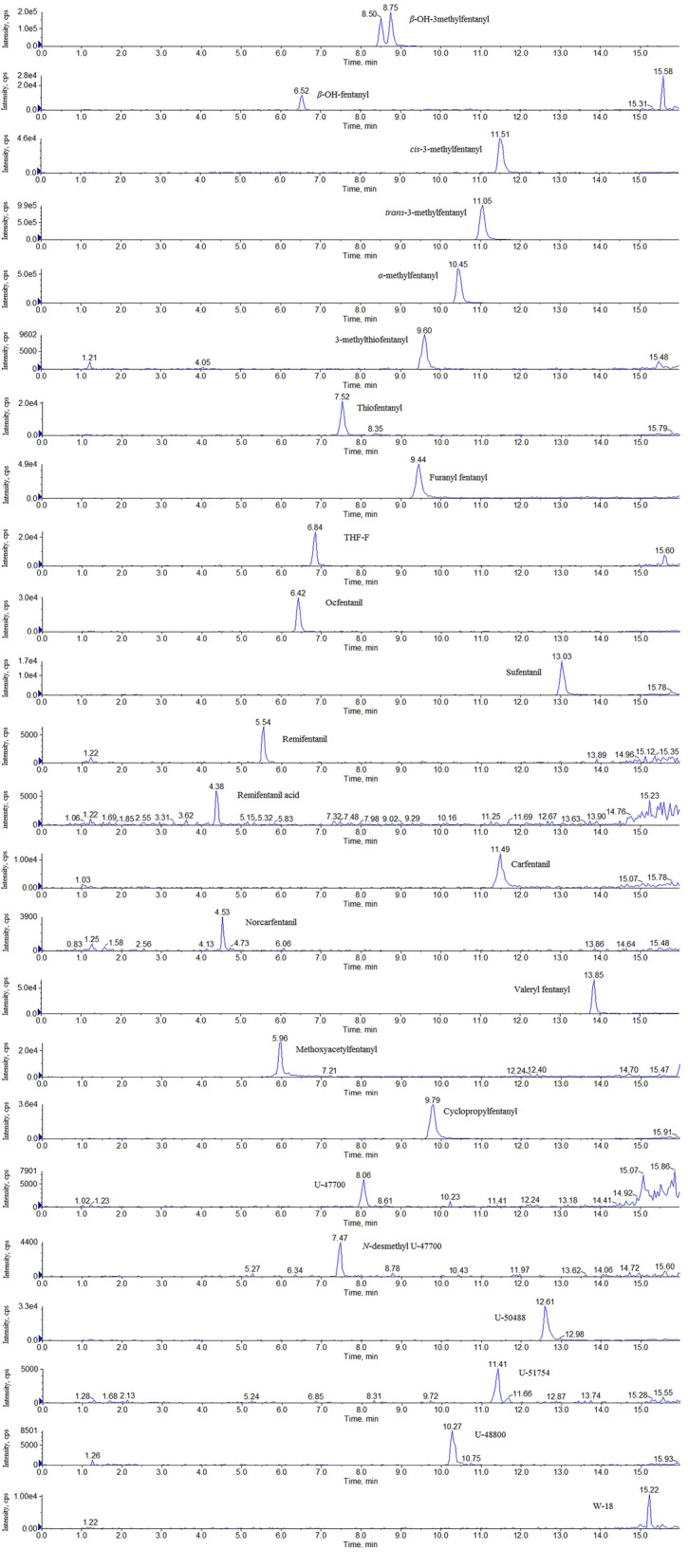
MRM chromatograms for the 37 analytes in hair samples spiked at the LOQ concentration.

No interfering signals were observed at the retention times of the analytes and ISs. Furthermore, there was no interference in blank hair spiked with common drugs of abuse and pharmaceuticals. The retention times are summarized in Table 2 and the chromatograms of all the analytes in hair samples spiked at the LOQ concentration are shown in Fig. 2.

#### Linearity, LOD, and LOQ

Table 3 summarizes the LOD, LOQ, regression equation, and R^2^ value obtained for each analyte. To the best of our knowledge, this is the first method for quantifying 31 fentanyl analytes and 6 novel synthetic opioids. The LODs for all the compounds ranged from 0.5 to 2.5 pg/mg, and the LOQs ranged from 2 to 5 pg/mg. Busardò et al*.*^25^ reported a method to quantify 22 fentanyl analogues in hair with LODs of 3–7 pg/g and LOQs of 11–21 pg/g. The calibration curves of all the analytes were established in different concentration range, but with acceptable correlation coefficients (R^2^ > 0.99). According to previous reports^3,25,41,43,44^, the concentrations of fentanyl analogues in hair samples are typically in the following ranges: 3–2,800 pg/mg for fentanyl, 15.1–149 pg/mg for norfentanyl, and 3–104 pg/mg for 4-ANPP. In addition, a concentration of 44 pg/mg has been reported for furanyl fentanyl^41^. Therefore, the linearity ranges obtained for these compounds in our study cover the ranges observed in authentic cases.

**Table 3:** LODs, LOQs, and linearity for analytes in hair.

Analyte	Range (pg/mg)	Regression equation	R^2^	LOD (pg/mg)	LOQ (pg/mg)
Fentanyl	2–2,500	y = 0.00243x + 0.00006	0.99672	0.5	2
Norfentanyl	5–2,500	y = 0.00779x—0.02772	0.99461	1	5
Alfentanil	2–2,500	y = 0.00235x + 0.00216	0.99417	0.5	2
Acetyl fentanyl	2–2,500	y = 0.00172x + 0.00205	0.99541	0.5	2
Acetyl norfentanyl	5–2,500	y = 0.00266x—0.00285	0.99204	2.5	5
4-ANPP	2–2,500	y = 0.00169x—0.00106	0.99664	0.5	2
Acryl fentanyl	2–2,500	y = 0.00164x + 0.00072	0.99698	0.5	2
Butyryl fentanyl	2–2,500	y = 0.00213x + 0.00100	0.99481	0.5	2
Isobutyryl fentanyl	5–2,500	y = 0.00194x + 0.00203	0.99566	2.5	5
PFBF	5–2,500	y = 0.00209x + 0.00050	0.99606	2.5	5
4-Fluoroisobutyryl fentanyl	5–2,500	y = 0.00173x + 0.00023	0.99688	2.5	5
*para/ortho*-Fluorofentanyl	5–2,500	y = 0.00151x—0.00028	0.99069	2.5	5
*β*-Hydroxythiofentanyl	5–2,500	y = 0.00041x + 0.00020	0.99740	1	5
*β*-Hydroxy-3-methylfentanyl	5–2,500	y = 0.00048x—0.00103	0.99788	1	5
*β*-Hydroxyfentanyl	5–2,500	y = 0.00056x—0.00094	0.99776	1	5
*cis*-3-Methylfentanyl	5–2,500	y = 0.00147x—0.00024	0.99636	1	5
*trans*-3-Methylfentanyl	5–2,500	y = 0.00202x—0.00499	0.99710	2.5	5
*α*-Methylfentanyl	5–2,500	y = 0.00175x—0.00335	0.99744	2.5	5
3-Methylthiofentanyl	5–2,500	y = 0.00074x—0.00121	0.99756	1	5
Thiofentanyl	5–2,500	y = 0.00127x—0.00217	0.99676	2.5	5
Furanyl fentanyl	5–2,500	y = 0.00243x + 0.00709	0.99379	1	5
THF-F	2–2,500	y = 0.00229x—0.00457	0.99379	0.5	2
Ocfentanil	2–2,500	y = 0.00271x + 0.00234	0.99345	0.5	2
Sufentanil	2–2,500	y = 0.00236x + 0.00121	0.99618	0.5	2
Remifentanil	2–2,500	y = 0.00062x + 0.00012	0.99564	0.5	2
Remifentanil acid	5–2,500	y = 0.00020x + 0.00024	0.99606	2.5	5
Carfentanil	2–2,500	y = 0.00098x + 0.00033	0.99648	0.5	2
Norcarfentanil	2–2,500	y = 0.00018x + 0.00093	0.99499	0.5	2
Valeryl fentanyl	5–2,500	y = 0.00251x + 0.00265	0.99686	2.5	5
Methoxyacetylfentanyl	2–2,500	y = 0.00224x + 0.00752	0.99222	0.5	2
Cyclopropylfentanyl	5–2,500	y = 0.00223x—0.00015	0.99537	1	5
U-47700	2–2,500	y = 0.00045x + 0.00022	0.99632	0.5	2
*N*-Desmethyl U-47700	2–2,500	y = 0.00166x + 0.00111	0.99714	0.5	2
U-50488	5–2,500	y = 0.00198x—0.00018	0.99570	1	5
U-51754	2–2,500	y = 0.00053x + 0.00025	0.99744	0.5	2
U-48800	2–2,500	y = 0.00063x + 0.00044	0.99505	0.5	2
W-18	5–2,500	y = 0.00028x + 0.00028	0.99606	2.5	5

#### Precision and accuracy

The precision and accuracy obtained for each analyte are listed in Table 4. The intraday and interday precisions of the all compounds at the LOQ, low, medium, and high levels were less than 13.32%. Furthermore, the accuracies at the four levels ranged from 85.63% to 116.1%, except for acetyl norfentanyl at the LOQ, which had an accuracy of 116.1%. Thus, the precision and accuracy of the method are acceptable according to the previous criteria^34–36^.

**Table 4:** Precision and accuracy for analytes in hair.

Analyte	Intraday precision (%)				Interday precision (%)				Accuracy (%)			
	LOQ	Low	Medium	High	LOQ	Low	Medium	High	LOQ	Low	Medium	High
Fentanyl	6.75	8.11	5.44	2.7	6.90	8.28	5.35	4.86	108.51	108.36	103.27	99.84
Norfentanyl	4.65	4.88	5.01	4.61	8.03	8.13	4.61	3.86	109.58	105.92	101.23	100.38
Alfentanil	7.92	6.52	6.5	3.99	12.41	7.48	4.82	3.83	85.63	104.7	106.06	101.36
Acetyl fentanyl	11.41	8.36	3.95	3.18	10.83	6.21	5.79	4.59	85.63	105.54	109.51	101.21
Acetyl norfentanyl	10.01	8.34	4.86	1.85	7.92	7.72	4.70	4.49	116.1	99.25	101.53	99.05
4-ANPP	9.07	6.12	2.85	4.85	6.25	6.52	6.29	6.45	106.1	96.39	107.11	107.41
Acryl fentanyl	10.96	6.74	3.48	5.9	8.02	6.74	4.70	4.19	101.79	98.45	106.38	100.99
Butyryl fentanyl	9.77	4.24	0.8	1.69	6.67	6.86	3.43	3.37	104	102.39	103.22	104.62
Isobutyryl fentanyl	7.04	5.93	3.27	5.02	7.85	6.56	7.74	4.92	89.43	101.87	102.89	105.05
PFBF	6.87	6.42	5.46	5.19	6.29	5.87	5.70	4.90	102.4	100.86	99.79	103.77
4-Fluoroisobutyryl fentanyl	9.92	9.62	2.26	4.98	7.43	9.12	6.17	6.10	108.5	102.76	103.51	104.88
*β*-Hydroxythiofentanyl	3.22	7.94	4.71	7.47	7.76	6.80	6.10	7.79	104.86	100.45	98.55	98.42
*β*-Hydroxy-3-methylfentanyl	4.23	2.25	4.47	4.38	6.77	4.49	7.16	4.74	110.02	96.88	101.18	100.17
*β*-Hydroxyfentanyl	4.83	5.56	5.02	7.28	4.98	4.51	5.50	6.43	105.54	100.59	103.82	107.32
*cis*-3-Methylfentanyl	5.09	5.23	1.94	6.03	5.19	6.06	5.99	4.59	107.87	101.41	99.45	101.03
*trans*-3-Methylfentanyl	2.8	3.03	5.01	4.16	3.40	4.30	4.40	4.80	105.19	96.17	98.95	100.84
*α*-Methylfentanyl	5.07	3.02	4.56	4.14	4.56	4.65	3.79	3.78	110.41	91.13	99.23	100.06
3-Methylthiofentanyl	2.95	1.91	3.58	6.02	4.33	4.80	3.67	4.62	110.81	99.35	106.51	104.22
Thiofentanyl	6.09	4.72	3.44	5.91	4.63	4.92	4.51	4.75	105.2	92.69	102.99	102.74
Furanyl fentanyl	6.15	7.39	3.01	6.72	6.64	5.66	5.87	6.69	100.96	102.99	108.86	105.52
THF-F	4.44	5.36	3.47	3.5	5.03	4.59	4.40	4.72	110.95	101.61	103.62	101.17
Ocfentanil	11.7	6.93	7.58	5.7	10.38	8.29	5.70	7.45	98.09	103	104.67	101.08
Sufentanil	6.37	5.48	3.74	6.24	11.29	7.34	6.50	7.87	98.46	100.68	101.87	102.07
Remifentanil	9.22	2.13	4.92	5.45	11.36	7.15	8.38	7.77	94.64	105.61	104.54	101.45
Remifentanil acid	13.32	8.74	5.85	5.56	11.16	8.72	5.55	7.51	105.74	95.1	98.6	100.23
Carfentanil	12.39	5.55	4.92	2.92	9.91	7.41	4.02	4.81	90.39	97.95	100.06	99.77
Norcarfentanil	10.77	10.5	3.19	5.25	11.10	10.66	5.42	5.20	113.53	101.54	107.1	103.53
Valeryl fentanyl	4.13	6.05	4.45	4.66	8.31	7.81	5.40	5.61	89.72	101	103.43	100.64
Methoxyacetylfentanyl	3.77	7.09	5.3	4.5	7.10	6.67	4.71	7.14	93.35	105.46	107.1	105.88
Cyclopropylfentanyl	3.53	3.19	2.73	3.76	4.78	6.63	4.03	3.28	107.34	100.67	98.91	98.22
U-47700	12.16	4.25	5.25	5.61	9.39	6.21	4.73	5.10	98.62	100.51	102.36	101.83
*N*-Desmethyl U-47700	5.62	4.01	2.66	4.57	10.96	7.42	3.02	5.40	96.15	99.63	103.01	94.64
U-50488	3.95	3.03	4.29	5.93	6.64	5.96	6.83	4.83	105.99	98.37	103.56	101.37
U-51754	5.27	3.1	3.91	5.13	10.23	7.26	3.59	6.37	103.18	99.89	102.3	103.91
U-48800	13.03	7.02	5.72	5.18	9.84	5.77	6.11	5.51	93.97	100.42	106.7	99.45
W-18	13.21	10.33	4.88	5.5	10.77	7.57	6.33	5.83	101.57	97.32	100.65	103.14

#### Recovery and ME

The extraction recovery and ME data are summarized in Table 5. The recoveries of all the analytes from the QC samples at four levels ranged from 89.42 to 119.68%. The ME values were within the range of 85.76–119.77%, except for that of W-18, which was in the range of 44.81–54.11%. Chromatographic evaluation of this compound was less than optimal owing to large fluctuations during elution.

**Table 5:** Recovery and matrix effect for analytes in hair.

Analyte	Recovery (%)								ME (%)							
	LOQ		Low		Medium		High		LOQ		Low		Medium		High	
	Mean	RSD	Mean	RSD	Mean	RSD	Mean	RSD	Mean	RSD	Mean	RSD	Mean	RSD	Mean	RSD
Fentanyl	101.13	2.25	100.46	3.92	96.97	3.30	97.70	3.68	96.22	10.27	104.87	2.85	101.19	0.89	99.98	0.98
Norfentanyl	89.42	8.66	98.20	1.84	93.21	5.18	95.65	1.54	103.58	8.23	111.70	4.46	103.76	3.35	98.65	2.44
Alfentanil	104.92	3.28	97.03	2.88	99.66	1.81	102.63	2.28	89.76	1.11	100.10	1.85	102.79	1.88	101.39	1.26
Acetyl fentanyl	99.71	2.53	97.55	3.31	96.61	34.88	98.68	3.26	89.04	5.97	103.15	3.71	102.58	1.75	102.23	2.92
Acetyl norfentanyl	105.26	3.91	101.71	1.48	93.61	1.21	96.45	4.58	90.29	3.86	99.71	1.65	99.77	4.61	95.73	1.31
4-ANPP	117.51	6.26	108.75	3.61	91.27	1.33	99.27	1.20	101.03	2.95	99.48	3.69	106.65	1.80	101.22	1.66
Acryl fentanyl	105.81	5.85	98.98	2.36	93.67	5.17	102.38	1.73	101.81	6.30	114.60	0.94	105.55	5.80	99.80	1.34
Butyryl fentanyl	102.76	5.71	102.92	4.40	96.50	2.83	101.51	2.54	102.66	1.52	114.55	1.87	98.36	3.36	98.65	2.58
Isobutyryl fentanyl	107.99	3.78	98.95	3.71	97.26	4.50	97.08	1.41	102.30	8.01	114.22	1.26	100.38	3.58	101.39	0.42
PFBF	119.68	2.37	103.92	2.43	98.49	2.42	103.56	5.38	96.22	5.16	108.46	2.27	98.53	3.13	99.17	1.84
4-Fluoroisobutyrylfentanyl	114.19	1.70	104.27	3.17	98.45	3.85	101.86	3.63	96.84	7.04	111.47	2.73	98.22	2.30	96.58	1.54
*para/ortho*-Fluorofentanyl	90.18	6.73	92.34	7.95	102.69	6.89	96.76	2.44	104.27	9.86	101.97	9.18	100.88	5.08	103.43	1.68
*β*-Hydroxythiofentanyl	101.67	2.56	97.56	3.29	97.11	2.08	94.89	1.77	93.15	2.41	93.53	5.83	87.96	3.08	93.94	0.85
*β*- Hydroxy -3-methylfentanyl	101.23	5.05	96.40	2.81	97.27	3.27	97.69	4.37	90.82	5.00	85.76	5.91	103.85	2.80	87.92	2.50
*β*-Hydroxyfentanyl	103.74	4.33	102.28	0.78	96.71	3.85	100.26	4.23	97.03	5.22	105.91	3.52	101.48	3.30	101.01	5.13
*cis*-3-Methylfentanyl	97.13	6.05	103.30	7.94	95.77	4.03	97.71	2.75	96.74	5.70	98.17	7.47	98.43	1.96	96.65	2.56
*trans*-3-Methylfentanyl	103.51	2.49	97.90	3.04	98.46	4.46	100.57	1.32	101.86	2.74	99.50	1.33	100.96	4.62	96.16	1.73
*α*-Methylfentanyl	111.37	9.80	102.26	3.83	97.66	3.48	97.37	2.88	96.41	4.15	95.26	3.85	96.27	1.86	100.68	0.97
3-Methylthiofentanyl	112.12	10.23	103.29	2.84	96.60	2.52	100.28	3.16	100.63	3.43	101.23	4.06	98.96	2.18	97.81	2.79
Thiofentanyl	110.10	5.11	100.93	4.24	107.86	13.89	99.35	4.62	92.38	4.44	103.19	4.72	93.52	5.20	92.40	2.73
Furanyl fentanyl	110.10	5.11	102.24	5.57	94.31	2.75	97.93	2.56	92.38	4.44	100.59	7.00	99.07	2.77	97.40	4.23
THF-F	99.50	6.28	98.76	1.85	101.49	1.72	98.83	2.60	93.05	5.30	98.57	1.53	99.00	2.62	98.53	2.55
Ocfentanil	98.26	3.19	100.31	3.23	92.29	6.01	95.65	7.61	97.24	3.45	105.83	3.40	100.60	2.66	101.45	3.53
Sufentanil	115.14	6.13	103.72	3.31	97.61	2.52	100.24	4.93	102.33	3.51	107.71	2.06	101.35	2.74	100.22	2.75
Remifentanil	102.07	4.82	99.43	5.45	93.51	3.11	98.46	2.49	94.44	1.72	99.40	6.45	96.13	2.78	94.31	2.32
Remifentanil acid	97.37	3.38	93.18	7.88	99.04	3.17	99.90	5.00	98.84	3.18	92.91	2.77	96.63	4.13	94.53	5.58
Carfentanil	106.17	4.90	102.49	1.82	95.61	0.79	97.66	3.36	95.56	5.83	102.04	2.03	100.98	2.32	98.33	9.81
Norcarfentanil	94.30	4.10	104.96	1.89	98.19	3.43	94.26	5.33	101.33	8.52	110.18	5.22	101.94	3.38	103.13	4.58
Valery fentanyl	89.83	6.31	94.43	2.78	111.60	2.53	97.50	4.63	103.85	7.85	102.27	2.49	104.05	2.67	104.27	4.64
Methoxyacetylfentanyl	98.28	9.42	103.41	2.85	99.82	2.18	99.81	2.15	89.09	10.49	100.55	5.07	96.22	2.30	95.54	2.06
Cyclopropylfentanyl	114.07	3.10	100.58	4.23	97.59	5.07	97.11	1.13	86.08	3.82	109.97	5.22	103.33	2.42	102.24	1.87
U-47700	98.69	5.48	105.55	7.59	98.79	5.24	100.99	1.76	99.45	5.09	112.99	2.37	102.58	4.13	98.98	0.98
*N*-Desmethyl U-47700	97.88	2.83	95.94	2.56	95.71	1.10	98.80	2.31	98.76	9.14	104.26	1.93	99.86	1.68	98.59	2.85
U-50488	92.42	5.05	108.03	7.22	101.49	1.72	105.96	1.80	99.42	11.22	119.77	0.83	99.00	2.62	96.25	1.07
U-51754	110.20	3.64	103.70	1.50	94.32	2.52	101.10	2.13	91.94	6.34	94.73	1.57	100.26	2.62	99.71	1.53
U-48800	105.69	5.20	110.81	12.41	97.22	2.57	100.49	3.82	92.65	3.76	99.91	3.66	104.05	1.84	95.47	2.01
W-18	99.08	6.21	99.27	6.95	98.58	4.68	102.12	6.15	50.03	5.44	54.11	8.50	45.78	8.87	44.81	12.35

#### Stability

The stability results for each analyte are shown in Table 6. The stabilities at the three concentration levels were within the range of 77.44–113.71% for all the analytes after storage in the autosampler at 4 °C for 24 h. Therefore, the developed method is suitable for use in daily forensic work.

**Table 6:** Stability of analytes in hair.

Analyte	Stability (%)			
	LOQ	Low	Medium	High
Fentanyl	97.41	98.40	102.22	99.20
Norfentanyl	96.95	91.44	100.11	98.88
Alfentanil	97.36	106.46	104.90	96.00
Acetyl fentanyl	99.38	98.75	96.95	95.58
Acetyl norfentanyl	109.88	97.86	96.29	98.03
4-ANPP	99.74	98.13	104.95	98.24
Acryl fentanyl	93.52	101.45	102.09	106.81
Butyryl fentanyl	103.25	97.48	99.75	99.97
Isobutyryl fentanyl	101.34	101.2	103.63	100.57
PFBF	99.11	97.48	96.86	93.8
4-Fluoroisobutyryl fentanyl	98.81	102.74	99.67	98.09
*para/ortho*-Fluorofentanyl	98.19	96.45	103.96	98.2
*β*-Hydroxythiofentanyl	101.12	94.18	95.9	101.09
*β*-Hydroxy-3-methylfentanyl	110.42	92.81	96.81	99.59
*β*-Hydroxyfentanyl	110.42	92.47	101.43	101.88
*cis*-3-Methylfentanyl	102.26	97.34	97.57	98.4
*trans*-3-Methylfentanyl	98.74	92.26	94.77	97.15
*α*-Methylfentanyl	103.91	98.36	99.49	99.4
3-Methylthiofentanyl	102.36	94.42	98.5	97.35
Thiofentanyl	103.96	89.7	99.76	101.41
Furanyl fentanyl	100.32	95.27	98.13	96.38
THF-F	113.71	77.44	102.77	102.19
Ocfentanil	96.87	102.53	97.51	97.07
Sufentanil	103.28	96.23	90.65	94.73
Remifentanil	109.76	103.29	103.83	105.56
Remifentanil acid	112.86	100.51	85.93	100.84
Carfentanil	108.82	99.51	99.48	100.2
Norcarfentanil	111.1	107.71	96.18	89.73
Valeryl fentanyl	102.76	101.94	91.56	96.27
Methoxyacetylfentanyl	101.73	95.8	96.57	98.1
Cyclopropylfentanyl	100.27	101.27	100.39	100.62
U-47700	95.03	101.17	101.21	100.85
*N*-Desmethyl U-47700	107.92	96.88	96.95	99.82
U-50488	106.58	102.37	95.95	98.66
U-51754	97.86	100.47	102.78	99.12
U-48800	108.75	102.16	99.67	101.82
W-18	101.96	93.04	104.82	85.09

### Application to authentic cases

**Figure 3:**
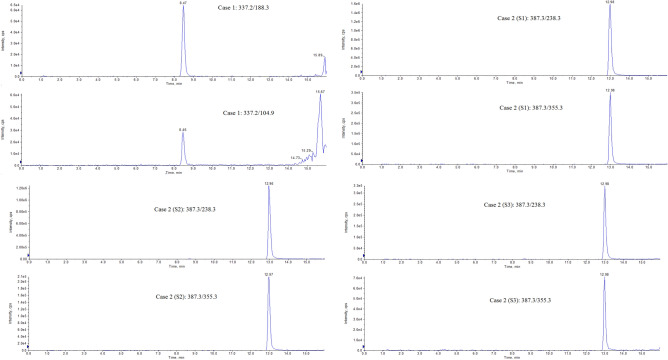
Chromatograms of two MRM transition in the authentic cases.

Following validation, the developed method was applied to the determination of fentanyl and its analogues in hair from authentic cases. The MRM chromatograms of cases 1 and 2 are shown in Fig. 3.

#### Case 1

A 35-year-old female patient without a history of drug abuse underwent surgery for thyroid disease. Anesthesia induction was performed by endotracheal intubation during surgery. One month after the operation, the patient's hair was collected and then the 0–3 cm segment of the hair sample was analyzed. In this case, fentanyl was detected at a concentration of 8.02 pg/mg. Schneider et al.^44^*.*reported a case in which a patient with a chronic and heavy toothache was treated with a fentanyl patch for 22 consecutive days. For a 5 cm hair sample cut into segments 0–1 cm, 1–2 cm, 3–4 cm, and 4–5 cm, the fentanyl concentration in all the segments was in the range of 60 pg/mg (LOQ) to 480 pg/mg. Compared with multiple doses, the concentration of fentanyl in hair was lower after a single dose, which may be why norfentanyl was not detected in our real case.

#### Case 2

A 51-year-old man was reported to police by his colleague. According to the informant, the man may have been using drugs for a long time. After hair collection, the hair sample was cut into three segments (S1: 0–3 cm, S2: 3–6 cm, and S3: 6–9 cm). Then, these samples were analyzed using our proposed method. Sufentanil was detected in the hair sample at concentrations of 183.91, 131.68, and 31.48 pg/mg for S1, S2, and S3, respectively. However, no metabolites were detected in the hair sample owing to the parent drugs being largely incorporated inside the keratin matrix from sweat, the bloodstream, and the sebum before metabolization. In this case, the observed concentration of sufentanil in hair will provide a reference for future forensic work.

## Conclusions

In this study, a sensitive, simple, rapid, and robust UHPLC-MS/MS was developed and validated for determination of 31 fentanyl analogues and 6 novel synthetic opioids in hair samples. This method covers fentanyl analogues and novel synthetic opioids that are common or new to the drug market. Furthermore, the developed method was successfully applied to authentic cases.
